# Pericapsular nervegroup (PENG) block—a scoping review

**DOI:** 10.1186/s42077-022-00227-0

**Published:** 2022-03-14

**Authors:** Gagandeep Kaur, Priyam Saikia, Samarjit Dey, Nayana Kashyap

**Affiliations:** 1 Gauhati Medical College and Hospital, Bhangagarh, Guwahati, Assam 781032 India; 2 All India Institute of Medical Sciences, Raipur, atibandh, Raipur, Chhattisgarh 492099 India

**Keywords:** Pericapsular nerve group block, PENG block, Scoping review

## Abstract

The obturator nerve and its accessory branch supplying the hip region, unlike the femoral nerve, are not adequately anaesthetized by conventional regional anaesthetic techniques targeting the hip region. With advancements in ultrasound-guided regional anaesthetic techniques, interests are revived in the field and efforts are being made to block them together. One such successful attempt is the novel peri-capsular nerve group (PENG) block. Consequently, further research on the PENG block began to explore its versatilities. The present study undertakes a scoping review of research on the PENG block in humans for any indication to determine the extent of the research done, the methodologies used, and other practical issues addressed in the research. This will help to identify potential research gaps that should be addressed in the future. PubMed, Google Scholar, Cochrane Library, KoreaMed, Latin American and Caribbean Health Science Literature, and the directory of open access journals were searched with the keyword “PENG block” and “pericapsular nerve group”. Manual search of electronically retrieved papers was also carried out. Among the 67 articles selected in our review, eighteen are case series; twenty-nine are case reports; two are randomized studies; one each of prospective cohort study, review, and cadaveric study; and 15 are editorials. However, only 64 articles were included later due to the retraction of three case reports. Data is insufficient to provide firm recommendations for or against different aspects of the block. The review encourages future work addressing different aspects of the PENG block.

**Supplementary Information:**

The online version contains supplementary material available at 10.1186/s42077-022-00227-0.

## Background

The use of ultrasound in regional anaesthesia has opened new targets for the provision of perioperative anaesthesia and analgesia (Albrecht & Chin, 2020). Description of fascial plane blocks has generated great enthusiasm, both inside and outside of the operation theatre (Albrecht and Chin, 2020). Its use is regarded as a standard clinical practice by many (Albrecht & Chin, 2020). The use of regional anaesthetic techniques will enjoy further acceptance as we have started to learn that its benefits are carried on well beyond the immediate postoperative period (Albrecht & Chin, 2020).

Pelvic fracture and surgery of the hip are very painful (Luftig et al., 2020). Less invasive peripheral nerve blocks including fascia iliaca compartment block (FIB) and femoral nerve (FN) block have shown considerable advantages as an analgesic modality and are being preferred in the analgesic and anaesthetic management of hip pathologies (Girón-Arango et al., 2018). However, these blocks inadvertently spare the obturator nerve (ON) and provide only moderate analgesia (Swenson et al., 2015; Guay & Kopp, 2020). Since articular branches of FN, ON, and accessory obturator nerve (AON) mainly innervate the anterior hip, there is a need for an easy, effective, and safe regional technique adequately targeting these structures at once (Short et al., 2018). Pericapsular nerve group (PENG) block has been proposed to address this need (Girón-Arango et al., 2018). This block was first described in late 2018 and has gained popularity among enthusiasts of regional anaesthesia (Girón-Arango et al., 2018; Yu et al., 2019; Ince et al., 2020). Considering the nascent stage of this technique, we intend to explore the literature on this topic.

### Rationale

Since the introduction, regional anaesthesia practitioners are intrigued by it and several case series (CS), case reports (CR), and editorials/commentaries and few randomized trials (RCT) have been published. With this review, we intend to examine, summate the latest advancements in the field, and identify the critical knowledge gap.

We conceived and started our review in early 2020 but it got delayed considerably due to the still prevalent COVID-19 pandemic. By the time we concluded our literature search, one review was published (Morrison et al., 2021). As we conceived this review to systematically retrieve available literature on PENG block, and the published review examined the role of PENG block in hip fracture and surgery only, we decided to proceed ahead with our review.

### Objectives

The review was conducted with the intention to systematically probe and summarize existing literature on the PENG block as well as to identify, if any, potential gap in the knowledge.

## Methods

### Ethics

As this is a review of published literature, we did not seek permission from the Institutional Ethics Committee.

### Protocol

Protocol was based on methodological steps outlined in the Arksey and O’Malley framework and further enhanced by Levac et al. and drafted using the Preferred Reporting Items for Systematic Reviews and Meta-Analysis Extension for Scoping Reviews (PRISMA-Scr) statement and was further revised (Arksey & O’Malley, 2005; Levac et al., 2010; Tricco et al., 2018).

### Eligibility criteria

All studies on the PENG block in humans from the first description of this block till our last electronic search were eligible for inclusion. After the inceptive search on 23rd April 2020, subsequent searches were done on 6th May 2020, 10th October 2020, and 10th January 2021. As a few eligible articles were subsequently retracted during the peer review stage of this manuscript, they were excluded subsequently. The block was described recently and ours is a scoping review, thus eligibility basis was not limited to any specific study design. We intended to include publications on anatomical studies as well. The articles that were not peer reviewed, or published in non-English language, or available as abstract, or poster, or study protocols were not included.

### Information sources

To identify potentially relevant documents, a search of PubMed, Google Scholar, KoreaMed, Cochrane Library, Latin American and Caribbean Health Science Literature (LILACS), and Directory of Open Access Journal (DOAJ) was carried out. Keywords used for the search were “PENG block” and “pericapsular nerve group block” (Table 1). Further articles were identified by scanning the reference list of the articles found in the primary search of the above-mentioned databases.

**Table 1:** Search strategy and results of database search on 10th Jan 2021

Name of the database	Search strategy	Filter	Number of results
PubMed	(PENG Block) NOT(Peng [Author])		61
	Pericapsular nerve group block		50
Cochrane Library	PENG Block in Title Abstract Keyword	Custom range 2018 to 2021	40
	Pericapsular nerve group block in Title	Custom range 2018 to 2021	38
	Abstract		
	Keyword		
DOAJ	PENG Block		356
	Pericapsular nerve group block		9
Google Scholar	PENG Block	With the exact phrase anywhere in the article	137
		Between 2018 and 2021	
	Pericapsular nerve group block	With the exact phrase anywhere in the article	50
		Between 2018 and 2021	
LILACS	PENGBlock	Title, abstract, subject	2
	Pericapsular nerve group block	Title, abstract, subject	0
KoreaMed	PENG[ALL] and Block[ALL]		2
	Pericapsular[ALL] and nerve[ALL] and group[ALL] and block[ALL]		0

*DOAJ* Directory of Open Access Journals, *LILACS* Latin American and Caribbean Health Sciences Literature

### Search

The search strategy was devised by PS and databases were searched by PS and GK independently. After the final search on 10th January 2021, the search results were collated by PS and GK. Any disagreement was settled by SD. The final search strategy can be found in Table 1.

### Data charting process

A data-charting form was jointly developed by PS and GK to include relevant data from eligible studies. It was further streamlined in an iterative updating process. Screening and data collection were done on Excel (Microsoft) by GK and PS and verified by all the authors.

### Data items

If available, we extracted publication details (author(s), year of publication, journal, publication type (case report/case series/review etc.), case details (total number of cases included, age group, and type of pathology), procedural details (type of surgical intervention, drug or local anaesthetic used, use of other (if any) additional general or regional anaesthetic and analgesic modality, outcomes—benefits and adverse effects), methodological highlights of the technique, and other facts judged to be of relevance by the authors. Details are mentioned in Additional file 1.

### Synthesis of results

The results are presented as a narration of details of different aspects of research. Literature does not define age groups clearly. The review considers patients with age more than 65 as elderly as suggested by WHO and follows the definition stated in section 520(m)(6)(E)(i) of the FD&C Act (relating to humanitarian device exemptions for paediatric patients) for paediatric patients as patients who are 21 years of age or younger at the time of the diagnosis or treatment (World Health Organization, 2015; Medical devices, 2014). The definition of CR and CS are not clearly stated in the literature. As suggested by Zidan et al., we considered case descriptions involving patients less than or equal to 4 as CR and more than 4 as CS (Abu-Zidan et al., 2012).

### Consultation phase

The results of the literature review were formulated and discussed among the team members. Subsequently, the final data set was finalized for this review.

## Results

### Selection of sources of evidence

**Fig. 1:**
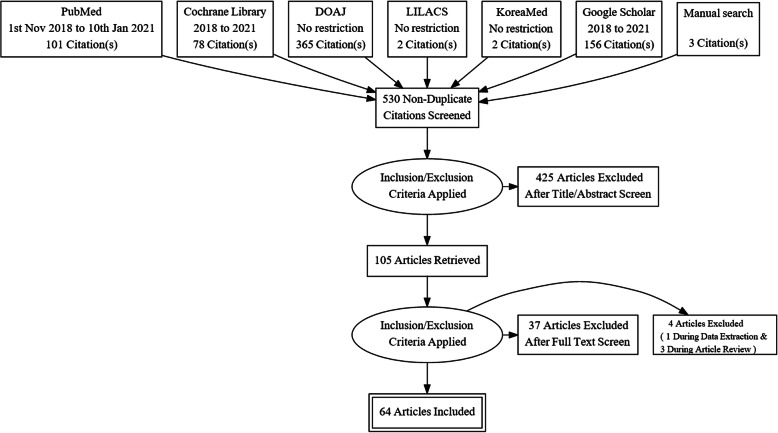
PRISMA (the Preferred Reporting Items for Systematic Reviews and Meta-Analysis Extension) flow diagram

After deletions of duplicates, the titles of a total of 530 citations were selected (Fig. 1). Among the 425 excluded titles, 406 were judged to be non-relevant to our review, and 19 were not in English. Among the 105 retrieved articles, 37 were excluded (book—1, poster—28, non-relevant—7, retracted article—1). One article was excluded during data extraction as it was in response to a retracted article. Among the 67 articles selected in our review, eighteen are CS, twenty-nine are CR, two are RCT, one each of PCS (prospective cohort study), review and cadaveric study, and 15 are editorials. In light of the latest changes, few retracted articles (3 CR) were excluded at the time of review of this manuscript, leaving a total of 64 articles to be included in the review. Apart from that, we identified 44 ongoing clinical trials.

### Original description of the block

**Fig. 2:**
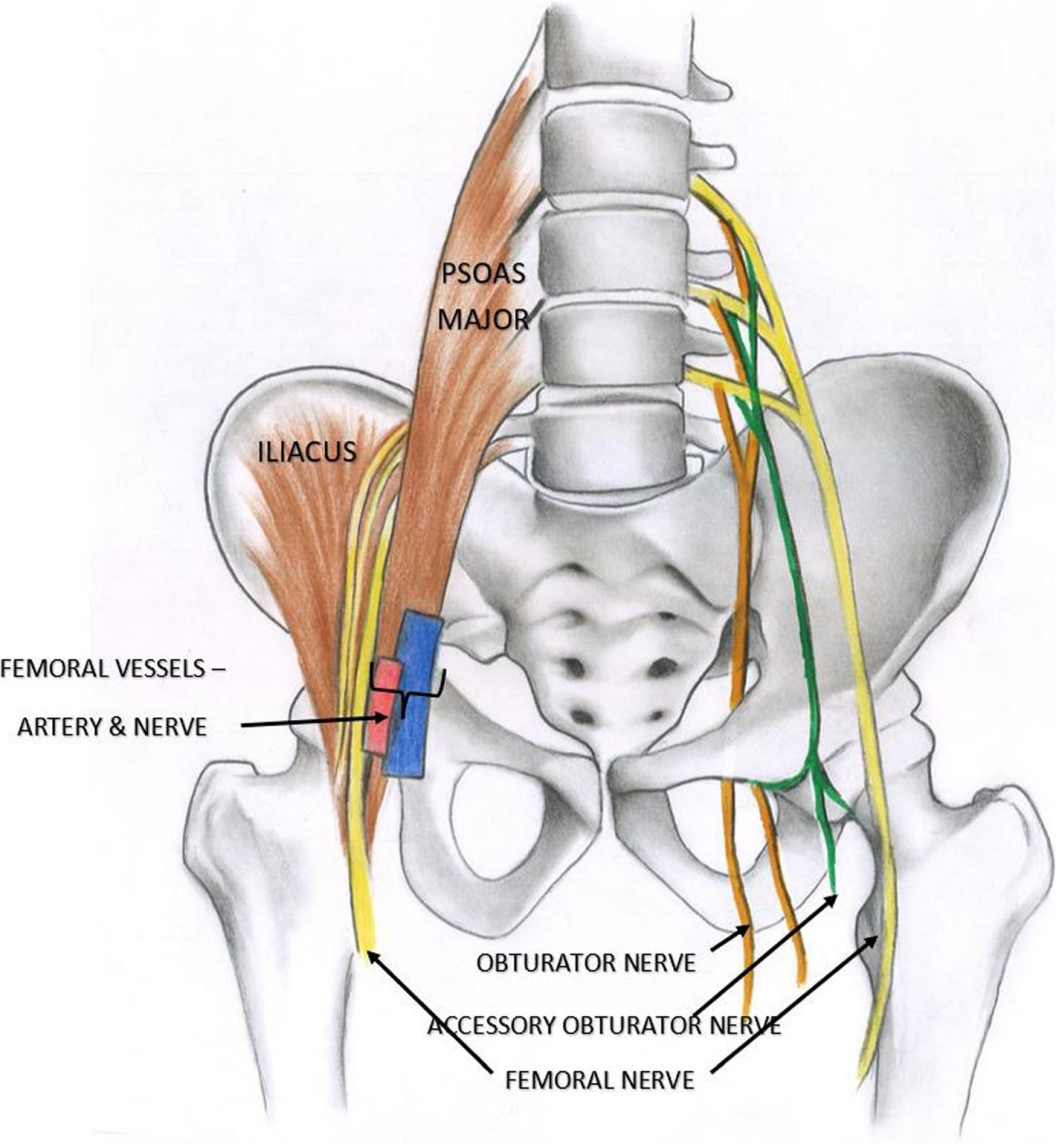
Branches of the lumbar plexus that contribute to innervation of the hip joint (right side of the picture) and their relation to the adjoining structures (left side of the picture)

The PENG block was first described by Girón-Arangoetal. as an ultrasound-guided regional anaesthetic approach focusing on adequate blockade of articular branches of the hip (Girón-Arango et al., 2018). It was based on anatomical studies which reported innervation of the hip capsule (Short et al., 2018). Literature suggests that the hip capsule is innervated by articular branches of the ON, AON, and FN (Fig. 2) and describes their relevant landmarks (Short et al., 2018). Girón-Arango et al. identified the musculo-fascial plane between the psoas tendon anteriorly and the pubic ramus posteriorly as the target area for the block (Girón-Arango et al., 2018). It was suggested that the articular branches of ON were blocked successfully due to the proximity of the target area to the subpectineal plane (Girón-Arango et al., 2018).

**Fig. 3:**
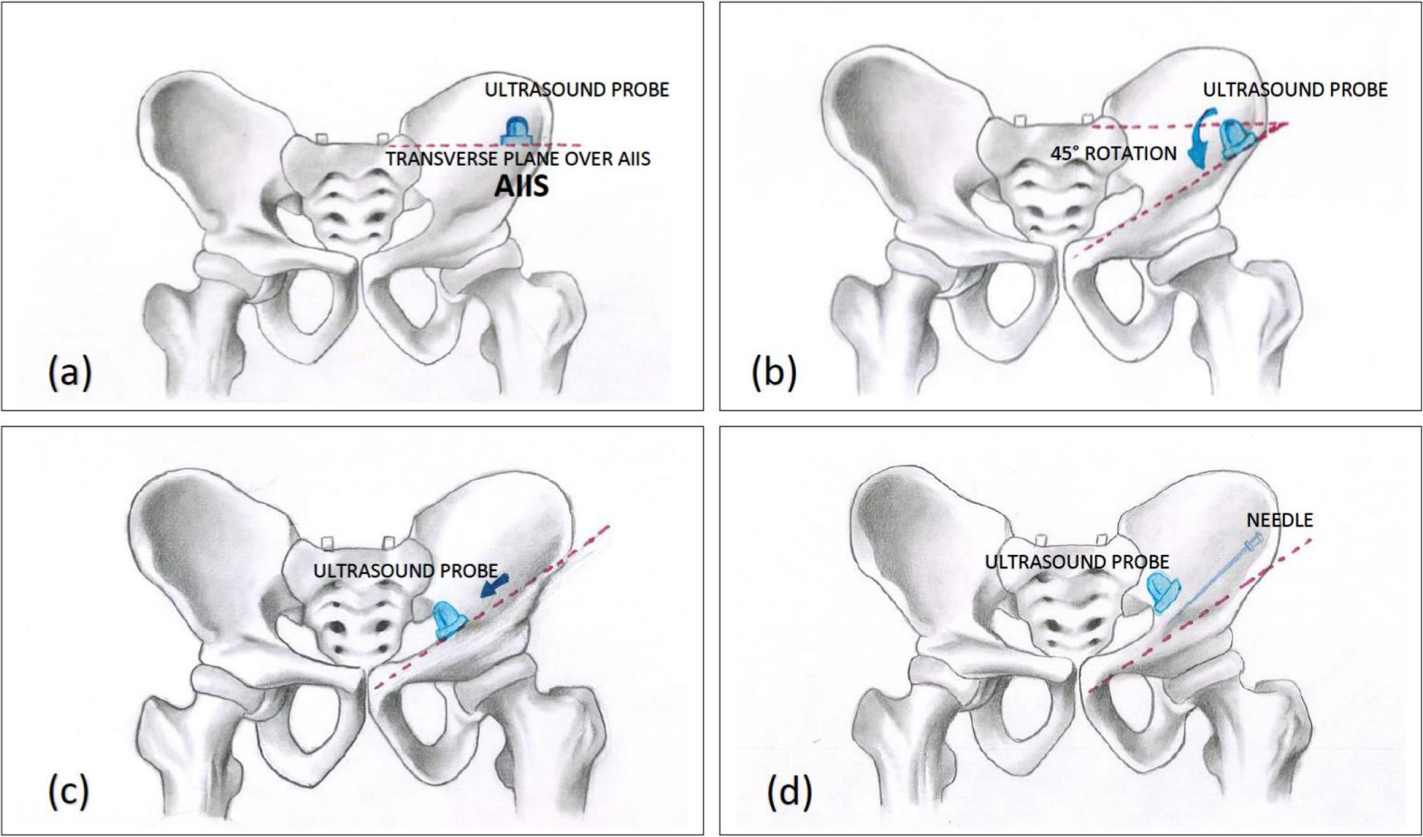
Steps to perform PENG block as described by Giron et al. **a** Ultrasound probe is placed in a transverse plane over the AllS. **b** Ultrasound probe is then aligned with the pubic ramus by rotating the probe approximately 45° (clockwise and counterclockwise respectively in right and left side of the patient). **c** Ultrasound probe is then positioned to observe the IPE, the iliopsoas muscle and tendon, and the femoral artery. **d** The needle is inserted from lateral to medial in an in-plane approach to place the tip in the musculofascial plane between the psoas tendon anteriorly and the pubic ramus posteriorly. Abbreviations: AIIS, anterior inferior iliac spine, IPE, ilio-pubic eminence

**Fig. 4:**
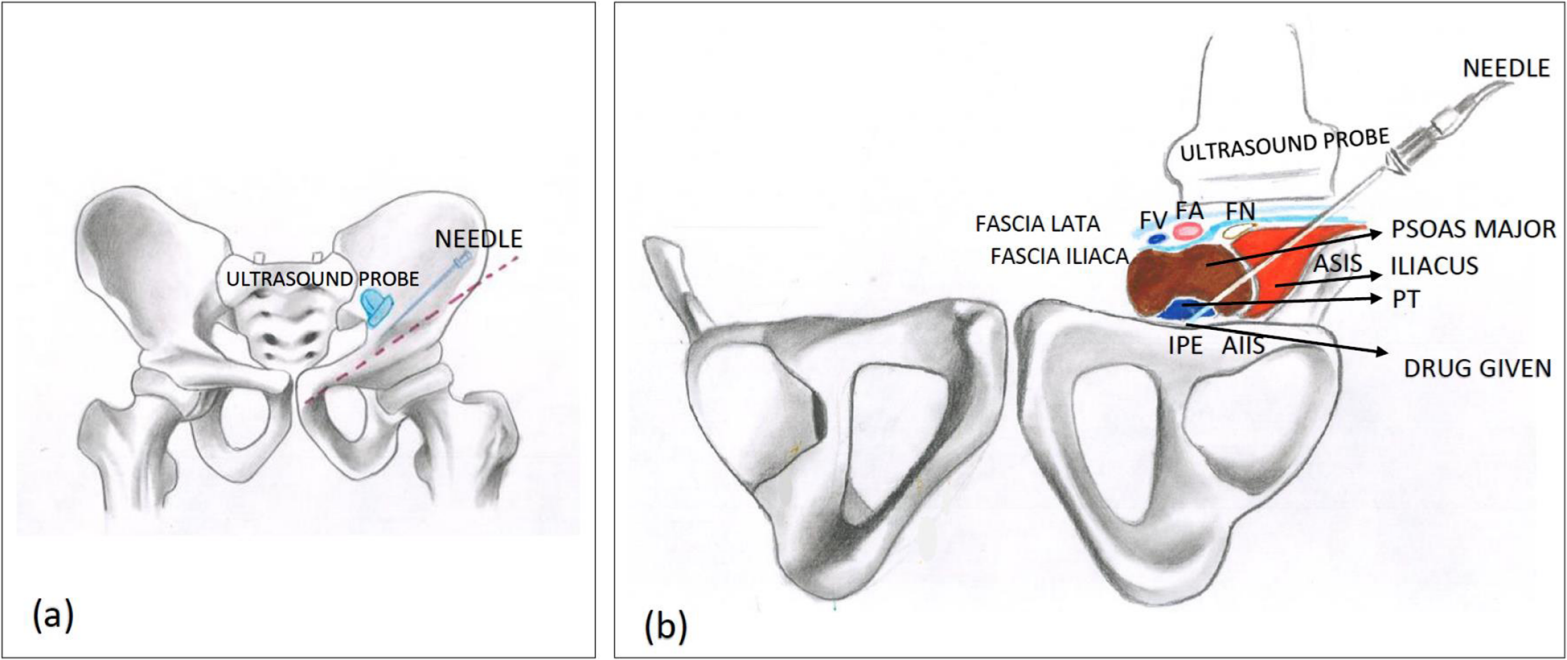
**a** Surface marking of the PENG block (standing position): The needle is inserted from lateral to medial in an in-plane approach while the ultrasound probe is positioned to observe the IPE, the iliopsoas muscle and tendon, and the femoral artery. **b** Pictorial representation of the PENG block (supine position): the needle is inserted from lateral to medial in an in-plane approach while the ultrasound probe is positioned to observe the IPE, the iliopsoas muscle and tendon, and the femoral artery. Abbreviations: ASIS, anterior superior iliac supine, AllS, anterior inferior iliac spine, IPE, ilio-pubic eminence, PT, psoas tendon, FN, femoral nerve, FA, femoral artery, FV, femoral vein

**Fig. 5:**
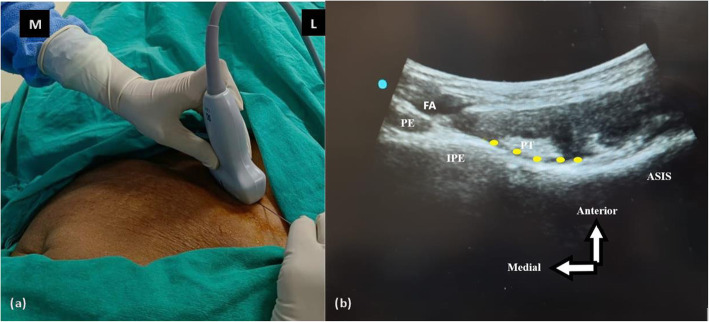
**a** Ultrasound guided PENG block is being given on the left side of the patient. Ultrasound probe is positioned with visualization of femoral vein, femoral artery, psoas tendon, iliacus fascia, and iliopubic eminence and the block needle is inserted in an in-plane approach from lateral to medial direction. **b** Illustration showing sonoanatomy of PENG block. In the view, femoral artery, psoas tendon, and iliopubic eminence are visualized. Local anaesthetic should be deposited in the area highlighted in yellow colour

In their description, a curvilinear (CL), low-frequency (LW) ultrasound probe was used with the patient in the supine position (Girón-Arango et al., 2018). The ultrasound probe was initially placed in a transverse plane over the anterior inferior iliac spine (AIIS) and then rotated counterclockwise approximately 45° (Girón-Arango et al., 2018). For right-sided block, clockwise rotation of the ultrasound probe is to be carried out. Before needle insertion, the iliopubic eminence, the iliopsoas muscle and tendon, the femoral artery, and the pectineus muscle were brought into view (Fig. 3) (Girón-Arango et al., 2018). The target area (the musculo-fascial plane between the psoas tendon anteriorly and the pubic ramus posteriorly) was approached, and 20 ml of drug volume was deposited with a 22-gauge needle along the plane of the ultrasound beam from lateral to medial (LTM) direction (Figs. 4 and 5) (Girón-Arango et al., 2018).

### Controversy surrounding the naming of the block

Amidst the criticism over the acronym “PENG”, which was also the last name of one of the authors of the index publication, even the phrase “PEricapsular Nerve Group” block drew attention over the intrinsic meaning of the term “pericapsular” (Girón-Arango et al., 2018; Nielsen & Bendtsen, 2021; Sidhu et al., 2020). It was unclear if the nomenclature was done so because the drug injected was distributed in the pericapsular space or it reflected the involvement of hip articular branches supplying the capsule per se. It was questioned if the acronym “APENG” is better suited as only nerves of the anterior capsule were blocked (Sidhu et al., 2020). However, since proprioceptive fibres constitute the main innervation of the posterior hip capsule, Peng et al. supported the use of the acronym PENG as blockade of nociceptive fibres in the anterior capsule was sufficient for the sensory block (Peng & Giron, 2019). Tran et al. supported the nomenclature in the view of their dye injection study which suggested distribution of the drug in the bursal space between the iliopsoas and anterior hip capsule (Tran et al., 2019). However, as administration of a higher volume of the drug has shown wider spread, the “pericapsular” characteristic of the block is still considered contentious (Ahiskalioglu et al., 2020a; Jadon et al., 2020a, b).

### Target site and spread of injectate

Numerous explanations have been proposed about the dissemination of the drug in the block. After injection into the target area (mainly involving articular branches of FN and AON) as in the initial description, spread to the subpectineal plane (containing articular branches of ON) is hypothesized to be by following the ilio-infratrochanteric muscular bundle throughout the “lacuna musculorum”(Girón-Arango et al., 2018;Pagano et al., 2019; Giron Arango & Peng, 2019; Nielsen et al., 2017; Fusco et al., 2019). The spread to ON was also supported in the view of the shorter course of the so-called barrier iliopectineal fascia in the cranio-caudal direction (Ahiskalioglu et al., 2020a). With the injection of a higher volume of the drug, PENG block has reportedly shown efficacy in acetabular and pelvic fracture, which may be due to the possibility of working as a hematoma block and spread of drug along the bone surface leading to sensory anaesthesia of the bone (Luftig et al., 2020). In the view of smaller potential space between iliopsoas eminence and psoas tendon, administration of higher volume of drug is believed to have leaked through deeper plane between the pectineus and the psoas to FN in the superficial plane (Giron Arango & Peng, 2019; Singh, 2021). The probability of leakage is believed to be higher with intramuscular injection and medial placement of the needle (Giron Arango & Peng, 2019; Girón-Arango et al., 2020). However, the rare involvement of the lateral femoral cutaneous nerve (LFCN) evincing to its 3-in-1 quality (FN, ON, and LFCN) is still unexplained (Santos et al., 2019). It is argued that the PENG block is an incomplete block as articular branches of the sciatic nerve innervating the posterolateral capsule of the hip joint are not captured by it (Sardesai & Biyani, 2020). Studies using dye in cadavers suggest that the drug spread to the posterior aspect of the hip thus covering the part of the sciatic nerve (Yamak Altinpulluk et al., 2020). There can be many anatomical rationales for the spread of the drug to different parts around the hip joint (Yamak Altinpulluk et al., 2020). Mistry et al. performed over 200 blocks, and based on their experience, they divided needle insertion areas into 3 zones and coloured them in white, grey, and black to present the risks associated with needle entry through them (Mistry & Sonawane, 2019). They identified the target site as white-coloured zone 1, superficial site near FN as black-coloured zone 2, and the area in between the two as grey-coloured zone 3 (Mistry & Sonawane, 2019). They advised to target the white zone and to avoid the black zone and in turn FN (Mistry & Sonawane, 2019). Though this description is helpful for the regionalist, the basis for the suggested zones is not clear. The published data is not sufficient to explain the anatomical facts of the target and adjacent area.

### Subsequent improvisations in the technique

Subsequent to the index description, many modifications in the technique have been suggested on the basis of individual experiences. The use of the curvilinear and low-frequency probe and “IN” plane approach was described originally; however, later studies demonstrated successful block while using the linear and high-frequency probe and out-of-plane approach (Girón-Arango et al., 2018; Ince et al., 2020; Jadon et al., 2020; Mistry & Sonawane, 2019; Shankar et al., 2020; Bilal et al., 2020; Aksu et al., 2020a; Orozco et al., 2019; Jadon et al., 2020; Ashok et al., 2020; Acharya & Lamsal, 2020). Black et al. recommend an easier way of identifying the target site by first visualizing the acetabulum or femoral head and then bringing the probe back proximally to visualize the transition to the linear contour of the iliopubic eminence (Black & Chin, 2019). Rotation of the needle between the thumb and index finger to facilitate the piercing of the fascial layer of the psoas muscle and to prevent intramuscular injection is suggested to prevent FN blockade (Giron-Arango & Peng, 2019).

Instead of the low-frequency probe, the use of high frequency is suggested for visualization of LFCN and in children and thinner built patients, considering a lower depth of the anatomical structures and better visibility (Mistry & Sonawane, 2019; Orozco et al., 2019; Ashok et al., 2020). In fact, studies suggested appropriate modifications considering the anatomical differences in adult and paediatric populations and male and female, e.g. 90° abduction of the hip and knee is suggested in paediatric patients for a better view (Aksu et al., 2020b).

A catheter has been placed between the iliopsoas muscle and the iliopubic eminence for continuous PENG block (Singh, 2021). Recently, the use of landmarks namely the anterior superior iliac spine (ASIS), pubic tubercle (PT), femoral artery (FA), and a point 5 cm medial to ASIS on the line joining ASIS and PT has been introduced in a landmark-guided PENG block technique (Jadon et al., 2020a, b). In the landmark-based technique, the needle insertion site is at the point 5 cm medial to ASIS with medial angulation, sufficient enough to avoid femoral artery puncture till bone is contacted (Jadon et al., 2020a, b). The use of a nerve stimulator is advocated to avoid FN block (Jadon et al., 2020a, b). Due to proximity to such vital anatomical structure, rigorous data about the safety of the landmark-based technique will be needed for wide acceptance.

Roy et al. reported additional analgesic benefit with the combination of LFCN and PENG block and Girón-Arango et al. suggested improvisation to give LFCN block in the same puncture by withdrawing the needle after giving the PENG block to the superficial plane between the fascia lata and the fascia iliaca (Roy et al., 2019;Girón Arango et al., 2019).

The published literature reports successful blocks with the use of the above-mentioned techniques. However, as no study has compared different technical aspects of the PENG block, any of these techniques is yet to be standardized.

### Combination and comparison with other anaesthetic modalities (regional and systemic techniques)

Pain and surgical management of hip pathologies involve one of the following blocks—lumbar plexus, FIB, LFCN, FN, sciatic nerve, quadratus lumborum (QL), lumbar erector spinae plane (LESP), PENG, and 3-in-1 block (Dangle et al., 2020). Studies have shown that the use of the above-mentioned blocks alone or in combination with other modalities results in adequate analgesia and reduction in opioid requirement (Dangle et al., 2020).

PENG block alone has been used successfully in various settings as an analgesic (Girón-Arango et al., 2018; Aydin et al., 2020). Shankar et al. compared PENG with FIB in a randomized manner, and though found them comparable in terms of duration of analgesia, significantly more reduction in pain score with PENG block was observed (Shankar et al., 2020).

Authors have argued if the iliopsoas plane (IP) and PENG block are similar (Nielsen & Bendtsen, 2019). It was pointed out that the target area of the PENG block is cranial to one for IP block and two are believed to be communicating with each other, rather than being two separate compartments (Nielsen & Bendtsen, 2021; Peng et al., 2019). Neilson et al. proposed blockade of all articular branches of FN by IP block unlike PENG block which was believed to spare the lower femoral branches leaving below the inguinal ligament (Nielsen & Bendtsen, 2019). Endersby et al. proposed the probability of lesser chances of motor block with IP block when compared to PENG block (Endersby et al., 2021). Further research to delineate the drug spread, distribution of nerve blockade, and contrast the clinical characteristics of both the blocks is warranted.

The combination of PENG and FN block was successful in providing acceptable analgesia in a paediatric patient with pain of the hip and thigh related to vaso-occlusive crisis (sickle cell disease) (Wyatt et al., 2020a, b). This combination was chosen based on the possible site of pain generators in that patient. The combination of PENG block and local anaesthetic infiltration (LIA) or PENG and lumbar erector spinae plane (LESP) block for postoperative pain after hip surgery has been used successfully (Ince et al., 2020; Fusco et al., 2019; Sandri et al., 2020; Ince & Kilicaslan, 2020). LFCN and PENG block and PENG and QL have also been successfully used in combination in hip surgery (Casas Reza et al., 2020; Kukreja et al., 2020a, b). In fact, the combination of PENG, FIB, and FN block has been reported to provide efficient analgesia while using 0.25% bupivacaine with a total of 70 (20 + 30 + 20) ml (Koyuncu et al., 2019). The use of such a high volume of local anaesthetic has however been questioned (Jadon, 2020).

In the light of increasing cases of inadvertent motor block, a reduction in the volume of drug administered in the PENG block has been stressed (Endersby et al., 2021).

Thus, there is a need to design studies to have a definite answer on the preferable block or an optimum combination of them for a particular surgery. Till then, understanding of the anatomy of pain generators, perioperative surgical plan, patient’s expectation, and perceived risk-benefit ratio should be considered on a case-by-case basis while formulating a procedural plan for regional analgesia/anaesthesia.

### Selection of infusate and dosing regimen

Local anaesthetics like bupivacaine, lidocaine, levobupivacaine, ropivacaine, and mepivacaine have all been used successfully in different concentrations, alone as well as in combination (Girón-Arango et al., 2018; Jadon et al., 2020a, b; Pagano et al., 2019; Aydin et al., 2020). Some researchers have also added dexamethasone and epinephrine to local anaesthetic (Luftig et al., 2020; Girón-Arango et al., 2018; Yu et al., 2019; Fusco et al., 2019; Orozco et al., 2019; Acharya & Lamsal, 2020; Wyatt et al., 2020a, b; Sandri et al., 2020; Fusco et al., 2020a, b; Mysore et al., 2020; Talawar et al., 2020; Ayub et al., 2020). Index description used 20 ml volume in the block and the use of a similar volume is reported in most of the subsequent publications (Girón-Arango et al., 2018). However, some used 10 ml and others preferred using a higher volume of 30 ml and 40 ml (Luftig et al., 2020; Ahiskalioglu et al., 2020a; Singh, 2021; Bilal et al., 2020; Aydin et al., 2020; Ayub et al., 2020; Romero et al., 2019; Remily et al., 2020; Ahiskalioglu et al., 2020a; Ahiskalioglu et al., 2020b). Speculating the potential space between the iliopsoas eminence and the psoas tendon to be small, Singh successfully used 10 ml volume while using the continuous technique with the intention to administer just enough volume for successful sensory block while avoiding chances of inadvertent motor block (Singh, 2021). Literature suggests the theoretical possibility of subpectineal obturator nerve block with the administration of a higher volume and proposed it as an alternative to lumbar plexus block (Ahiskalioglu et al., 2020a). Continuous PENG block, infusing local anaesthetic at the rate of 5ml/h and 7ml/h (believed to have added advantage of the distal femoralblock), has also been demonstrated to be an efficient technique (Singh, 2021; Santos et al., 2019; Wyatt et al., 2020a, b; Del Buono et al., 2020; Singh et al., 2020; Jacob Wolf, 2019; Prado-Kittel et al., 2020; Singh, 2020).

There are no good-quality data to evaluate the comparison and determine an optimal local anaesthetic solution, adjuncts, and their concentration and volume for the block.

### Indications

Initially, the PENG block was reported as an effective perioperative analgesic technique in adult patients with hip fracture (Girón-Arango et al., 2018). Later on, it has been used in elderly and paediatric patients and even in fragile co-morbid patients (Luftig et al., 2020; Yu et al., 2019; Ince et al., 2020; Santos et al., 2019; Shankar et al., 2020; Aksu et al., 2020a; Orozco et al., 2019; Wyatt et al., 2020a, b; Ince & Kilicaslan, 2020; Fusco et al., 2020a, b; Romero et al., 2019; Ahiskalioglu et al., 2020a, b; Wyatt et al., 2020a, b; Wolf, 2019; Singh, 2020; Thallaj, 2019; Fusco et al., 2020a, b; Jaramillo et al., 2020). Perioperatively, it has been used alone or in combination with general and other regional techniques as an analgesic or anaesthetic technique in open and arthroscopic hip surgery, hip positioning, acetabular fracture, pelvic fractures, surgery of medial thigh, vein ligation and stripping surgery, in the prevention of adductor muscle spasm during TURBT, and during the management of the acute phase of opioid- resistant hip vaso-occlusive crisis (Luftig et al., 2020; Shankar et al., 2020; Bilal et al., 2020; Orozco et al., 2019; Acharya & Lamsal, 2020; Aydin et al., 2020; Wyatt et al., 2020a, b; Sandri et al., 2020; Ayub et al., 2020; Ahiskalioglu et al., 2020a; Ahiskalioglu et al., 2020b; Prado-Kittel et al., 2020; Orozco et al., 2020; Alrefaey & Abouelela, 2020; Sahoo et al., 2020). Its successful use for postoperative analgesia, both with a single injection and continuous infusion, has also been reported (Fusco et al., 2019; Aksu et al., 2020a; Roy et al., 2019; Casas Reza et al., 2020; Mysore et al., 2020; Thallaj, 2019). The use of catheters has been suggested to further extend the duration of analgesia, while simultaneously minimizing the needed volume of drug and preventing rebound hyperalgesia (Singh, 2021; Wyatt et al., 2020a, b). In fact, its potential as the first step in embarking outpatient total hip arthroplasty in light of the reduction of pain, post-op medical and surgical complications, and thus duration of hospital stay has been mentioned (Remily et al., 2020). Even its use as a regional ablative technique for pain management has been explored with chemical and radiofrequency neurolysis (Romero et al., 2019;Jaramillo et al., 2020).

Due to the lack of good-quality data on the efficacy and safety of this block for a specific indication, no consensus can be drawn on the indication of this block. But considering the volume of reported analgesic outcomes in patients with a few specific hip surgeries, the role of PENG block is worth considering.

### Impact of the PENG block on outcomes

#### Advantages

The PENG block, like other regional anaesthetic techniques may offer effective perioperative analgesia (can be prolonged with the use of longer acting local anaesthetic, adjuvants, and catheter placement) and simultaneously reduce the requirement of opioids and related effects like nausea, vomiting, delirium, etc. (Girón-Arango et al., 2018; Pagano et al., 2019; Singh, 2021; Ayub et al., 2020). Being a primary sensory block, it may facilitate early mobilization and recovery, unlike other nerve blocks (Luftig et al., 2020; Yu et al., 2019; Pagano et al., 2019). Authors believe it to be comparatively safer in patients with comorbidity (Ahiskalioglu et al., 2020a; Dangle et al., 2020; Fusco et al., 2020a, b; Kukreja et al., 2020a, b). It has been pointed out not to consider it as a safe block in patients on anticoagulants in general, and its use in appropriate clinical context has been urged (Black & Chin, 2019). The use of the supine position makes block administration a bit easier technique for first-line therapy pain management or positioning for neuraxial anaesthesia (Luftig et al., 2020; Shankar et al., 2020; Acharya & Lamsal, 2020; Ayub et al., 2020; Romero et al., 2019; Del Buono et al., 2020; Fusco et al., 2020a, b; Jaramillo et al., 2020). It may contribute to lower drug usage and consequent hemodynamic stability during neuraxial anaesthesia (Fusco et al., 2020a, b).

### Complications

Authors opine that there is a probability of injury to anatomical structures in close relation to the target area. Mistry et al. suggested the possibility of injury of the ureter with more medial needle advancement (Mistry et al., 2019). However, Aksu et al. hinted towards injury of the bladder itself, instead of the ureter, and recommended to take the history of last urination and to do a negative aspiration test for urine to avoid the injury (Aksu et al., 2020b). Yu et al. reported motor block in the form of quadricep weakness and proposed the use of normal saline or small volumes (1–2 ml) of local anaesthetic for hydrolocation to identify the site of the needle tip (Yu et al., 2019). Intramuscular injection and medial needle placement were suggested to be the possible reasons for the spread to FN resulting in motor block and it was recommended to rotate the needle while piercing the fascia so as to avoid intramuscular injection (Giron Arango & Peng, 2019). Inadvertent intravascular placement of catheter can also ensue during continuous PENG block as experienced by Buono et al. for which medial re-placement was considered a plausible solution (Del Buono et al., 2020). Preliminary ultrasound scanning, local anaesthetic infiltration at the needle insertion site for warning signs of paresthesia, and adoption of out-of-plane (OUT) approach is speculated to decrease the likelihood of inadvertent LFCN injury (Jadon et al., 2020a, b;Ashok et al., 2020).

However, limited data is available on adverse effects, length of hospital stay, and other outcomes. Hence, a detailed assessment is needed in the future about the causation, prevention, and treatment measures for the associated complications.

## Discussion

### Summary of evidence

The published literature on the PENG block suggests that it has potential as an effective analgesic and anaesthetic technique perioperatively alone or in combination with other conventional techniques. Over time, literature has explored various aspects of the block like the approach, the amount of the volume of local anaesthetic needed, and its use in combination with other blocks as described in Table 2.

**Table 2:** Scope of literature

Publication detail		Detail
First author	Sl. no. as in Additional file 1	
Girón-Arango et al.	20	Described PENG block first as ultrasound guided regional anaesthetic approach
Jadon et al.	26	Introduced a landmark guided PENG block technique
Lufting et al.	34	Higher volume of drug was used
Black et al.	11	Recommends an easier way of identification of target site by first visualizing acetabulum or femoral head
Del Buono et al, Jacob wolf et al., Prado-Kittel et al., Santos et al., Singh et al., Wyatt K et al.	13, 24, 46, 52, 55, 56, 57, 61	Used continuous PENG block
K Shankar et al.	30	Compared PENG with FIB in a randomized manner and though found them comparable in terms of duration of analgesia, significantly more reduction in pain score with PENG block
Wyatt et al.	61	Used combination of PENG block and FN block
Fusco et al., Sandri et al., Mysore et al.	15, 38, 51	Used combination of PENG block and LIA block
Ince et al.	22, 23	Used combination of PENG block and LESP block
Roy et al., Thallaj et al., Talawar et al., Casas reza et al.	12, 43, 58, 59	Used combination of PENG block and LFCN block
Kukreja et al.	33	Used combination of PENG block and QL block

Abbreviations: *PENG* Pericapsular nerve group block, *FIB* Fascia iliaca compartment block, *FN* Femoral nerve, *LIA* Local anaesthetic infiltration, *LESP* Lumbar erector spinae plane; *LFCN* Lateral femoral cutaneous nerve, *QL* Quadratus lumborum

Data is not sufficient enough to provide firm recommendations for different aspects of the block. Many RCTs have been registered with the trial registry and the recent future may answer many knowledge gaps identified.

### Limitations

Majority of the published literature are retrospective and are case series, case reports, or commentaries based on personal experiences of practitioners. There is limited availability of prospective or randomized studies till date. This reflects a danger of oversimplification or overinterpretation.

### Conclusions

We have reviewed the available relevant data on the PENG block. Inferior quality data suggests PENG block as a possibly useful perioperative analgesic and anaesthetic technique, alone or in combination with other techniques depending upon the surgery, while decreasing opioid-related side effects. However, data is insufficient to provide firm recommendations on most of the aspects. Future cadaveric, anatomical, radiological, and clinical trials will be needed to determine detailed anatomical facts about the target and surrounding area, distribution and mechanism of the PENG block, nature of the spread of the drug, standard procedure for the technique, safest method of the needle insertion, optimal local anaesthetics, and their adjuncts, optimal concentration and volume of the drug in adults and children, optimal time to perform the block considering analgesic benefit and post-operative distortion of normal tissue planes and consequent adverse effects, etc. Another potential area of importance will be establishing its effectiveness in comparison to conventional techniques, in finding which surgical procedures gain benefit from the technique, in finding efficacy in ASA 3 and 4 patients and impact on outcomes like pain, adverse effects, length of hospital stay, etc. It was exciting to understand the development of this novel technique and it will be really exhilarating to follow future discoveries.

## Supplementary Information

**Additional file 1.** Data items.

## Data Availability

The data will be available upon reasonable request to the corresponding author.

## Abbreviations

AON: Accessory obturator nerve
AIIS: Anterior inferior iliac spine
ASIS: Anterior superior iliac spine
CR: Case reports
CS: Case series
DOAJ: Directory of open access journals
FIB: Fascia iliaca compartment block
FA: Femoral artery
FN: Femoral nerve
IP: Iliopsoas plane
IPE: Ilio pubic eminence
LILACS: Latin American and Caribbean Health Science Literature
LFCN: Lateral femoral cutaneous nerve
LIA: Local anaesthetic infiltration
LESP: Lumbar erector spinae plane
ON: Obturator nerve
PENG: Pericapsular nerve group
PCS: Prospective cohort study
PT: Pubic tubercle
QL: Quadratus lumborum
RCT: Randomized clinical trials
