# Supplementary material for: Pericapsular nervegroup (PENG) block—a scoping review
Source: Ain-Shams J Anesthesiol. 2022 Mar 14;14(1):29. doi: 10.1186/s42077-022-00227-0 (PMC8919174; doi:10.1186/s42077-022-00227-0)
Supplement: Supplementary file 1 — Additional file 1. Data items. [file 42077_2022_227_MOESM1_ESM.docx]

|  | PUBLICATION DETAIL | | | | PATIENT DETAIL | | | | | | INTERVENTION DETAIL | | | | | | | | | | DRUG DETAIL | | | | | | TECHNIQUE DETAIL | | | | | | |
| --- | --- | --- | --- | --- | --- | --- | --- | --- | --- | --- | --- | --- | --- | --- | --- | --- | --- | --- | --- | --- | --- | --- | --- | --- | --- | --- | --- | --- | --- | --- | --- | --- | --- |
| Serial no. | First Author | Authors | Journal/Book | Title | Type of report | Total number of cases | Age | Target Hip patholgy | Target Other pathology | Comorbidities | Type of surgical intervention | PENG | PENG Administration | Comparison/ Combination | Other block | Other anaesthetic technique | Other analgesics | Focus of management | Outcomes—efficiency | Outcomes—adverse effects | LA used | Concentration used in % | Volume used in ml | Adjuvant | Epinephrine | If single/continous used | Probe type | Frequency Type | Frequency exact values in MHz | Approach—IN/OUT | Approach LTM /MTL | If single/continous used | Position |
| 1 | Acharya U | Acharya U, Lamsal R. | Case Rep Anesthesiol | Pericapsular Nerve Group Block: An Excellent Option for Analgesia for Positional Pain in Hip Fractures | CS | 10 | P/ Ad/ El | Y | N | NM | Hip surgery | Y | Y | N | N | N | N | Pos_A | Reduction in pain score (NRS). The NRS score was 3 for three patients, 2 for four patients, and the rest had a score of 1 during active movement 10 minutes after block.The NRS score was 2 for three patients, 0 for one patient, and the rest had a score of 1 after sitting upright during the administration of SAB | N | B | 0.125 | 20 | D | N | S | CL | LW | NM | OUT | NM | S | Supine |
| 2 | Ahiskalioglu A | Ahiskalioglu A, Aydin ME, Ahiskalioglu EO, Tuncer K, Celik M. | J Clin Anesth | Pericapsular nerve group (PENG) block for surgical anesthesia of medial thigh | CR | 1 | Ad | N | Mass in medial thigh | Y | Mass excision | Y | Solo | N | N | 1 mg midazolam | N | IOA | Uneventful perioperative period with no use of additional analgesic. | N | B,LD | 0.5,2 | 30 (15+15) | N | N | S | CL | NM | NM | IN | NM | S | NM |
| 3 | Ahiskalioglu A | Ahiskalioglu A, Aydin ME, Ozkaya F, Ahiskalioglu EO, Adanur S. | J Clin Anesth | A novel indication of Pericapsular Nerve Group (PENG) block: Prevention of adductor muscle spasm | CR | 2 | Ad/ El | N | Adductor muscle spasm in TUR-BT | NM | TURBT | Y | After GA/SA | N | N | GA/SA | N | Prevention of adductor muscle spasm | No adductor muscle spasm | N | B,LD | 0.5,2 | 30 (15+15) | N | N | S | CL | NM | NM | IN | NM | S | Lithotomy / Supine |
| 4 | Ahiskalioglu A | Ahiskalioglu A, Aydin ME, Celik M, Ahiskalioglu EO, Tulgar S. | J Clin Anesth | Can high volume pericapsular nerve group (PENG) block act as a lumbar plexus block? | CR | 2 | Ad/ El | Y | Y | NM | Vein ligation and stripping and Positioning for SA | Y | Solo | N | N | N | N | Pos_A POA | Sensory testing of required dermatomes revealed suffiecient level of block. No additional analgesia needed | Significant quadriceps weakness reported in one patient | B,LD | 0.5,2 | 30 (15+15) | N | N | S | NM | NM | NM | NM | NM | S | NM |
| 5 | Aksu C | Aksu C, Cesur S, KuÅŸ A. | J Clin Anesth | Pericapsular nerve group (PENG) block for postoperative analgesia after open reduction of pediatric congenital dysplasia of the hip | CR | 1 | P | Y | N | NM | Hip surgery | Y | After GA | N | N | GA | Fentanyl (preoperatively), PCM and ibuprofen (postoperatively) | IOA POA | Reduction in pain score ( NRS) | N | B | 0.25 | 10 | N | N | S | Lin | HH | 10 to 18 | IN | LTM | S | Supine |
| 6 | Aksu C | Aksu C, Cesur S, KuÅŸ A. | J Clin Anesth | Pericapsular Nerve Group (PENG) block: Controversial points about anatomical differences | EDITORIALS |  |  |  |  |  |  |  |  |  |  |  |  |  |  |  |  |  |  |  |  |  |  |  |  |  |  |  |  |
| 7 | Alrefaey AK | Alrefaey AK, Abouelela MA | Egyptian Journal of Anaesthesia | Pericapsular nerve group block for analgesia of positioning pain during spinal anesthesia in hip fracture patients, a randomized controlled study | RCT | 60 | Ad | Y | N | N | Hip surgery | Y | Before SA | Comparison-with and without PENG | N | SA | N | Pos_A | The PENG block was associated with a statistically significant lower pain and comfortable positioning during spinal anaesthesia levels compared to the control group. | N | B | 0.25 | 20 | N | N | S | NM | HH | NM | IN | NM | S | Supine |
| 8 | Aydin ME | Aydin ME, Borulu F, Ates I, Kara S, Ahiskalioglu A. | J Cardiothorac Vasc Anesth | A Novel Indication of Pericapsular Nerve Group (PENG) Block: Surgical Anesthesia for Vein Ligation and Stripping | CR | 2 | Ad | N | Varicose vein | NM | Vein ligation and stripping | Y | Solo | N | N | 1 mg midazolam, Propofol infusion | 50 ug fentanyl | IOA | Uneventful perioperative period with no use of additional analgesic except for 1 mg midazolam and 50 ug fentanyl and proopofol infusion. | N | B,LD | 0.5,2 | 30 (15+15) | N | N | S | CL | NM | NM | IN | NM | S | NM |
| 9 | Ayub A | Ayub A, Bhoi D, Tangirala N, Narayanan MV | Indian Journal of Pain | Initial experience of pericapsular nerve group block for positioning during neuraxial block in patients with hip fracture. | CS | 5 | El | Y | N | NM | Hip surgery | Y | Before SA | N | N | SA | Paracetamol | Pos_A POA | Reducion in pain score (NRS) from 8 (before block) to 3 (15 minutes after block) and 4 (at the time of positioning for SA). Comfortable positioning for SA. Postoperative median time to first request of analgesia was 4 h. | N | R,LD | 0.5,2 | 10 (5+5) | N | Y | S | CL | LW | 2 to 5 | IN | LTM | S | Supine |
| 10 | Bilal B | Bilal B, Ã–ksÃ¼z G, Boran Ã–F, Topak D, DoÄŸar F. | J Clin Anesth | High volume pericapsular nerve group (PENG) block for acetabular fracture surgery: A new horizon for novel block | CR | 2 | Ad | Y | N | NM | Hip surgery | Y | After GA | N | N | GA | paracetamol 1 g and ibuprofen 400 mg (intraoperatively) and PCM (Postoperatively) | IOA POA | Uneventful perioperative period and Reduction in pain score (NRS) to 2 and 3 in first and second case at 24 h respectively | N | B | 0.25 | 30 | N | N | S | Lin | HH | NM | IN | NM | S | Supine |
| 11 | Black ND | Black ND, Chin KJ. | J Clin Anesth | Pericapsular nerve group (PENG) block: Comments and practical considerations | EDITORIALS |  |  |  |  |  |  |  |  |  |  |  |  |  |  |  |  |  |  |  |  |  |  |  |  |  |  |  |  |
| 12 | Casas Reza P | Casas Reza P, Diéguez García P, Gestal Vázquez M, Sampayo Rodríguez L, López Álvarez S | Minerva Anestesiol | Pericapsular nerve group block for hip surgery | CS | 8 | El | Y | N | Y | Hip surgery | Y | After SA/GA | N | LFCN | GA/SA | Dexketoprofen and paracetamol | POA | Reduction in postoperative morphine consumption | N | LB | 0.375 | 20 | N | N | S | CL | LW | 1 to 5 | NM | LTM | S | Supine |
| 13 | Del Buono R | Del Buono R, Padua E, Pascarella G, Soare CG, Barbara E. | Reg Anesth Pain Med | Continuous PENG block for hip fracture: a case series | CS | 10 | El | Y | N | N | Hip surgery | Y | Before SA | N | N | SA | PCM | Pos_A POA | Reduction in pain score (NRS) from 7 to 2 , 20 minutes after PENG block. The median pain score (NRS) after 12,24,48 h were 2. | N | R(S), LD(S), R© | 0.375(S), 0.5(S), 0.2© | 20 (S) + 5 ml/h© | N | N | S + C | NM | NM | NM | NM | NM | S + C | Supine |
| 14 | Endersby RV | Endersby RV, Moser JJ, Ho EC, Yu HC, Spencer AO | Acta Anaesthesiologica Scandinavica | Motor blockade after iliopsoas plane (IPB) and pericapsular nerve group (PENG) blocks: A little may go a long way | EDITORIALS |  |  |  |  |  |  |  |  |  |  |  |  |  |  |  |  |  |  |  |  |  |  |  |  |  |  |  |  |
| 15 | Fusco P | Fusco P, Di Carlo S, Paladini G, Scimia P, Di Martino E, Marinangeli F, Petrucci E. | Reg Anesth Pain Med | Could the combination of PENG block and LIA be a useful analgesic strategy in the treatment of postoperative pain for hip replacement surgery? | CR | 4 | Ad | Y | N | N | Hip surgery | Y | Before SA | Combination with LIA | LIA | SA | Acetaminophen | POA | Reduction in pain score - rest and dynamic pain score were 2 and 4 controls during first 24 h after surgery. SUGGESTED use of multimodal therapy for postoperative analgesia | N | LB | 0.375 | 20 | D | N | S | CL | LW | 3 to 5 | IN | NM | S | NM |
| 16 | Fusco P | Fusco P, Petroni GM, Ciccozzi A, Tullj S, Chiavari R, Marinangeli F. | Minerva Anestesiol | The role of the PENG block in hip fracture in El patient with severe comorbidities | CR | 1 | El | Y | N | Y | Hip surgery | Y | Before SA | N | N | SA | N | IOA POA | Reduction in dose of LA used for SA. Haemodynamically stable and Uneventful perioperative period. | N | LB | 0.375 | 15 | N | N | S | CL | LW | 3 to 5 | NM | NM | S | Supine |
| 17 | Fusco P | Fusco P, De Sanctis F, De Paolis V, Di Carlo S, Di Martino E, Volpe D, Chiavari R, Petrucci E, Ciccone A, Marinangeli F. | Minerva Anestesiol | Could US-guided PENG block pass the ICU door? First reported case in a multiple-fracture ICU patient | CR | 1 | El | Y | N | Y | Pain management | Y | After Sedation | N | N | Dexmedetomidine sedation | N | Analgesia | Reduction in pain score (NRS) from 8 before block to 4 and 1 at rest and dynamic at 24 h. | N | LB | 0.375 | 20 | D | N | S | CL | LW | 3 to 5 | IN | NM | S | Supine |
| 18 | Giron Arango L | Giron Arango L, Peng P. | Reg Anesth Pain Med | Reply to Dr Yu et al: Inadvertent quadriceps weakness following the pericapsular nerve group (PENG) block | EDITORIALS |  |  |  |  |  |  |  |  |  |  |  |  |  |  |  |  |  |  |  |  |  |  |  |  |  |  |  |  |
| 19 | Girón-Arango | Girón-Arango L, Tran J, Peng PW. | J Cardiothorac Vasc Anesth. | Reply to Aydin et al.: A Novel Indication of Pericapsular Nerve Group Block: Surgical Anesthesia for Vein Ligation and Stripping. | EDITORIALS |  |  |  |  |  |  |  |  |  |  |  |  |  |  |  |  |  |  |  |  |  |  |  |  |  |  |  |  |
| 20 | Giron-Arango L | Giron-Arango L, Peng PWH, Chin KJ, Brull R, Perlas A. | Reg Anesth Pain Med | Pericapsular Nerve Group (PENG) Block for Hip Fracture | CS | 5 | Ad/ El | Y | N | N | Hip surgery | Y | NM | N | N | N | N | Analgesia | Reduction in NRS pain score - Rest and dynamic -at 30 min - median reduction was 7 points | N | B,R | 0.25,0.5 | 20 | D | Y | S | CL | LW | 2 to 5 | IN | LTM | S | Supine |
| 21 | Giron-Arango L | Giron-Arango L, RoquÃ©s V, Peng P. | Reg Anesth Pain Med | Reply To Dr Roy et al: Total postoperative analgesia for hip surgeries: PENG block with LFCN block | EDITORIALS |  |  |  |  |  |  |  |  |  |  |  |  |  |  |  |  |  |  |  |  |  |  |  |  |  |  |  |  |
| 22 | Ince I | Ince I, Kilicaslan A. | J Clin Anesth | Combination of Lumbar Erector Spinae Plane Block (LESP) and Pericapsullar Nerve Group (PENG) block in hip surgery | CR | 3 | El | Y | N | NM | Hip surgery | Y | Before SA | Combination-PENG and LESP block | LESP block | SA | Fentanyl with PCA and 1 g PCM | Pos_A POA | Reduction in pain score and sensory testing of required dermatomal level showed efficient block. | N | B,LD | 0.5,2 | 20 (10+10) | N | N | S | NM | NM | NM | NM | NM | S | Supine |
| 23 | Ince I | Ince I, Kilicaslan A, Kutlu E, Aydin A. | J Clin Anesth | Combined pericapsular nerve block (PENG) and lumbar erector spinae plane (ESP) block for congenital hip dislocation surgery | CR | 1 | P | Y | N | N | Hip surgery | Y | After GA | Combination of PENG and LESP block | LESP | GA | Paracetamol | POA | Pain score (FLACC score ) at 1, 2, 4, 6, 12, 24 h were 0, 1, 1, 0, 0, 0, respectively. | N | B | 0.25 | 8(PENG) +12 (LESP) | N | N | S | Lin | NM | NM | IN | LTM | S | Supine |
| 24 | Jacob Wolf | Jacob Wolf | Int Student J Nurse Anesth | Pericapsular Nerve Group block in the Total Hip Arthroplasty Patient | CR | 1 | El | Y | N | Y | Hip surgery | Y | After surgery | N | N | Propofol | Acetaminophen | POA | Reduction in pain score to 0-3 | N | B,LD,NS,R | 0.5,1,0.2 | 25(S) + 6 ml/h© | N | N | S + C | CL | NM | NM | NM | NM | S + C | Supine |
| 25 | Jadon A | Jadon A, Neelam S, Bhupendra S, Amit A | SF J Radiol Clin Diagn | Out-Of-Plane Approach to Prevent Injury to Lateral Femoral Cutaneous Nerve (LFCN) in Pericapsular Nerve Group (PENG) Block | CR | 4 | El | Y | N | N | Hip surgery | Y | Before SA | N | N | SA | N | Pos_A | Reduction in pain score (NRS) - rest and dynamic - from 7 and 9 to 2 and 4 respectively from before block to 30 minutes after block. Comfortable positioning during SA. | N | B | 0.25 | 20 | D | N | S | NM | LW | 2 to 5 | OUT | NM | S | Supine |
| 26 | Jadon A | Jadon A, Sinha N, Chakraborty S, Singh B, Agrawal A. | Indian J Anaesth | Pericapsular nerve group (PENG) block: A feasibility study of landmark based technique | CS | 10 | Ad/ El | Y | N | N | Hip surgery | Y | Y | Comparison of USG(4 patients) and Landmark (6 patients) guided PENG | N | SA | N | Pos_A | Reduction in pain score (NRS) - rest and dynamic - from 6 and 8 to 2 and 3. Comfortable positioning during SA. Median EOSP score was 3. | N | B | 0.25 | 20 | D | N | S | CL | LW | 2 to 5 | OUT | NM | S | Supine |
| 27 | Jadon A | Jadon A, Sinha N, Chakraborty S, Ahmad A | Bali Journal of Anesthesiology | An out-of-plane approach for pericapsular nerve group block: A case series | CS | 10 | Ad/ El | Y | N | N | Hip surgery | Y | Before SA | N | N | SA | N | Pos_A | Comfortable positioning while SA. Reduction in pain score (NRS) - rest and dynamic - from 6.5 to 3.8 and 8.5 to 5.1 before and 30 minutes after block respectively. Mean EOSP score was 2.6. | N | B | 0.25 | 20 | D | N | S | NM | LW | 2 to 5 | OUT | NM | S | Supine |
| 28 | Jadon A | Jadon A | Brazilian Journal of Anesthesiology | In response to- “Three Blocks including Pericapsular Nerve Block (PENG) for a femoral shaft fracture pain” by Onur Koyuncu et al. | EDITORIALS |  |  |  |  |  |  |  |  |  |  |  |  |  |  |  |  |  |  |  |  |  |  |  |  |  |  |  |  |
| 29 | Jaramillo S | Jaramillo S, Muñoz D, Orozco S, Herrera AM | J Clin Anesth | Percutaneous bipolar radiofrequency of the pericapsular nerve group (PENG) for chronic pain relief in hip osteoarthrosis | CR | 2 | El | Y | N | Y | Hip pain | Y | After Sedation | N | N | N | N | Analgesia | Reduction in pain score (VAS) from 7 and 10 in 2 patients to 0­—1 till 3 months | N | Bipolar radiofrequency ablation for 60 s at 80° | _ | _ | _ | _ | S | NM | NM | NM | NM | NM | S | Supine |
| 30 | K Shankar | K Shankar, Srinivasan Rangalakshmi, AB Ashwin, et al. | Indian J Anesth Analg | Comparative Study of Ultrasound Guided PENG [Pericapsular Nerve Group] Block and FIB [Fascia Iliaca Block] for Positioning and Postoperative Analgesia Prior to Spinal Anaesthesia for Hip Surgeries: Prospective Randomized Comparative Clinical Study. | RCT | 60 | Ad/ El | Y | N | Y | Hip surgery | Y | Before SA | Comparison ­— PENG and USG guided FIB | FIB (USG guided) | SA | Rescue analgesic — fentanyl and tramadol | Pos_A IOA POA | comfortable positioning while SA. Reduction in pain score (VAS) from 7.8 to 0.6 during positioning in PENG group block and from 7.6 to 2.6 during positioning in FI block. Comparable postoperative analgesia in both. | N | R | 0.25 | 25 | N | N | S | Lin | HH | 7 to 15 | NM | NM | S | Supine |
| 31 | Koyuncu O | Koyuncu O, Hakimoğlu S, Polat ST, Kora MY | Braz J Anesthesiol | Three blocks including Pericapsular Nerve Block (PENG) for a femoral shaft fracture pain | CR | 1 | Ad | Y | N | N | Hip surgery | Y | Before GA | PENG block — FIB — FN block — GA | FIB, FN block | GA | Tramadol, paracetamol | IOA POA | Reduction in pain score (NRS) from 10 (before block) to 7 ( after PENG block), to 5 (after FI Block) and to 4 (after FN block) | N | B | 0.25 | 20 +30 + 20 | N | N | S | NM | NM | NM | NM | NM | S | NM |
| 32 | Kukreja P | Kukreja P, Avila A, Northern T, Dangle J, Kolli S, Kalagara H. | Cureus | A Retrospective Case Series of Pericapsular Nerve Group (PENG) Block for Primary Versus Revision Total Hip Arthroplasty Analgesia | CS | 12 | Ad/ El | Y | N | N | Hip surgery | Y | Before SA and GA | N | N | SA/GA | N | Analgesia | Reduction in pain score (VAS) and cumulative oral morphine equivalent (OME) usage. Average postoperative opioid use in the first 24 h was 78.7 OMEs in the revision group, compared to 18.4 OMEs in the primary group . | N | R | 0.5 | 20 | N | N | S | CL | LW | NM | IN | LTM | S | Supine |
| 33 | Kukreja P | Kukreja P, Schuster B, Northern T, Sipe S, Naranje S, Kalagara H. | Cureus | Pericapsular Nerve Group (PENG) Block in Combination With the Quadratus Lumborum Block Analgesia for Revision Total Hip Arthroplasty: A Retrospective Case Series | CS | 16 | Ads/El | Y | N | NM | Hip surgery | Y | Before GA/SA | Comparison of QL block with combination of QL and PENG block | Quadratus lumborum block | GA/SA | N | POA | Significantly lower values of average pain scores at 6 and 24 h point in combination of PENG and QL block as compared to only QL block. The average oral morphine equivalents required in first 12 and 24 h were 66.1 and 90.7 mg in QL only group and 43.8 and 72.6 mg in the PENG nad QL group, respectively. | N | R | 0.5 | 20 | N | N | S | CL | LW | NM | IN | LTM | S | Supine |
| 34 | Luftig J | Luftig J, Dreyfuss A, Mantuani D, Howell K, White A, Nagdev A. | Am J Emerg Med | A new frontier in pelvic fracture pain control in the ED: Successful use of the pericapsular nerve group (PENG) block | CR | 3 | El | Pelvic fracture | N | N | Pain management | Y | Y | N | N | N | N | Analgesia | Pain relief. Able to range the hip movements with minimal or no pain. | N | B, NS | 0.5, 0.9 | 30 (20 +10) | N | Y | S | CL | LW | NM | IN | NM | S | Supine |
| 35 | Mistry T | Mistry T, Sonawane KB, Kuppusamy E. | Reg Anesth Pain Med | PENG block: points to ponder | CS | 5 | Ad | Y | N | N | Hip surgery | Y | NM | N | N | CSE / GA | N | Pos_A IOA POA | Reduction in pain score | No adverse effect reported. On the basis of experience suggested possibility of injury to ureter on medial needle advancement. | NM | NM | NM | NM | NM | S | NM | NM | NM | NM | NM | S | NM |
| 36 | Mistry T | Mistry T, Sonawane KB. | J Clin Anesth | Gray zone of pericapsular nerve group (PENG) block | CS | 200 | Ad/ El | Y | Y | NM | Various including hip surgery | Y | NM | NM | NM | NM | NM | Pos_A POA | Efficient analgesia. | N | B,R | 0.25,0.2 | 20 | N | N | S | CL,Lin | LW,HH | NM | IN,OUT | NM | S | NM |
| 37 | Morrison C | Morrison C, Brown B, Lin DY, Jaarsma R, Kroon H. | Reg Anesth Pain Med | Analgesia and anesthesia using the pericapsular nerve group block in hip surgery and hip fracture: a scoping review | REVIEW |  |  |  |  |  |  |  |  |  |  |  |  |  |  |  |  |  |  |  |  |  |  |  |  |  |  |  |  |
| 38 | Mysore K | Mysore K, Sancheti SA, Howells SR, Ballah EE, Sutton JL, Uppal V. | Can J Anaesth | Postoperative analgesia with pericapsular nerve group (PENG) block for primary total hip arthroplasty: a retrospective study | CS | 123 | Ad/ El | Y | N | NM | Hip surgery | Y (47 patients) | Y | Comparison ( Alone LIA with combination of PENG and LIA) | LIA | N | intravenous PCA hydromorphone, acetaminophen (3–4 g daily), and a nonsteroidal anti-inflammatory drug | IOA POA | Reduction in 24 r postoperative hydromorphone equivalent consumption after using PENG to 4.5 mg from 5.5 mg | N | B, NS | 0.25 | 20 | D | Y | S | CL | LW | NM | IN | LTM | S | Supine |
| 39 | Nielsen ND | Nielsen ND, Bendtsen TF. | Acta Anaesthesiol Scand. | In response: Motor blockade after iliopsoas plane (IPB) and pericapsular nerve group (PENG) blocks: A little may go a long way. | EDITORIALS |  |  |  |  |  |  |  |  |  |  |  |  |  |  |  |  |  |  |  |  |  |  |  |  |  |  |  |  |
| 40 | Nielsen ND | Nielsen ND, Bendtsen TF | Reg Anesth Pain Med | Motor-sparing regional analgesia for hip-derived pain | EDITORIALS |  |  |  |  |  |  |  |  |  |  |  |  |  |  |  |  |  |  |  |  |  |  |  |  |  |  |  |  |
| 41 | Orozco S | Orozco S, MuÃ±oz D, Jaramillo S, Herrera AM. | J Clin Anesth | Pediatric use of Pericapsular Nerve Group (PENG) block for hip surgical procedures | CR | 1 | P | Y | N | N | Hip surgery | Y | After GA | N | FN and LFCN block | GA | N | IOA POA | Uneventful perioperative period with no use of additional analgesic. Reduction in pain score to 2 after 72 h. | N | B | 0.5 | 10 | N | Y | S | Lin | HH | NM | NM | LTM | S | Supine |
| 42 | Orozco S | Orozco S, Munoz D, Jaramillo S, Herrera AM. | J Clin Anesth | Pericapsular Nerve Group (PENG) block for perioperative pain control in hip arthroscopy | CS | 5 | Ad | Y | N | NM | Hip surgery | Y | NM | Combination — FN block | FN block | GA | N | POA IOA | Uneventful perioperative period with no use of additional analgesic. Reduction in pain score (VAS) to 3 in recovery room and upto 24 h and to 0 or 1 after 48 - 72 h. | N | B,LD | 0.75,1 | 20(10+10) | N | N | S | CL | LW | NM | NM | NM | S | Supine |
| 43 | Pagano T, | Pagano T, Scarpato F, Chicone G, Carbone D, Bussemi CB, Albano F, Ruotolo F | Arthroplasty | Analgesic evaluation of ultrasound-guided Pericapsular Nerve Group (PENG) block for emergency hip surgery in fragile patients : a case series | CS | 6 | El | Y | N | Y | Hip surgery | Y | Before SA | N | N | SA | N | Pos_A IOA POA | Reduction In pain score (NRS) rest and dynamic - 4.83 and 6 points respectively. | N | R,M | 0.5,1 | 20 | N | N | S | CL | LW | 2 to 5 | IN | LTM | S | Supine |
| 44 | Peng P | Peng P, Giron Arango L. | Reg Anesth Pain Med | Reply to Dr Sidhu et al: APENG vis-à-vis PENG | EDITORIALS |  |  |  |  |  |  |  |  |  |  |  |  |  |  |  |  |  |  |  |  |  |  |  |  |  |  |  |  |
| 45 | Peng PWH | Peng PWH, Perlas A, Chin KJ. | Reg Anesth Pain Med | Reply to Dr Nielsen: Pericapsular Nerve Group (PENG) block for hip fracture | EDITORIALS |  |  |  |  |  |  |  |  |  |  |  |  |  |  |  |  |  |  |  |  |  |  |  |  |  |  |  |  |
| 46 | Prado-Kittel C | Prado-Kittel C, Zumelzu-Sánchez P, Palma-Licandeo A, Faúndez-Lillo G, Ellenberg-Oyarce K, Jorquera-Adarme B. | Revista Española de Anestesiología y Reanimación (English Edition | Continuous pericapsular nerve group blockade as analgesia for fracture of the posterior column and wall of the acetabulum; a case report and description of infusion regimen for extending analgesic effect to the distal femoral area. | CR | 1 | Ad | Y | N | Y | Pain management | Y | Solo | N | N | N | N | Analgesia | Reduction in pain score (VAS ) from 10 before block to EVA 3 at 5 min, 0 and 2(rest and dynamic) for 48 h, and 0 and 2 for additional 72 h. Continous PENG block successfully provided analgesia for 72 h with extention to distal femoral region with 7 ml/h infusion rate. | N | LB(S), LD(S) + B© | 0.25(S),1(S) + 0.1© | 20 (S) + 5 and 7 ml/h © for first 48 and 72 h resp. | N | N | S + C | NM | NM | NM | IN | LTM | S + C | Supine |
| 47 | Remily EA | Remily EA, Hochstein SR, Wilkie WA, Mohamed NS, Thompson JV, Kluk MW, Nace J, Delanois RE. | Hip Int | The pericapsular nerve group block: a step towards outpatient total hip arthroplasty? | CS | 96 | Ad/ El | Y | N | N | Hip surgery | Y | After surgery | Y | FI block | SA | Acetaminophen, NSAID, gabapentin | POA | Reduction in pain score, shorter time and increased distance of ambulation, longer therapeutic window before requiring first opioids, and shorter length of stay in hospital. | N | B | 0.5 | 10 | N | Y | S | Lin,CL | HH ,LW | 3 to 13 | NM | LTM | S | Supine |
| 48 | Romero AR | Romero AR, Valdy GC, Lemus AJ | Can J Anesth | Ultrasound-Guided pericapsular nerve group (PENG) hip joint phenol neurolysis for palliative pain. | CR | 1 | El | Y | N | Y | Hip pain | Y | Y | N | N | N | N | Neurolytic PENG block for pain management | Complete pain relief was obtained 30 min later and continued relief till 2 weeks and no motor weakness. | N | Phenol | 6 | 10 | N | N | S | CL | LW | 3 to 8 | IN | LTM | S | Supine |
| 49 | Roy R | Roy R, Agarwal G, Pradhan C, Kuanar D. | Reg Anesth Pain Med | Total postoperative analgesia for hip surgeries, PENG block with LFCN block | CS | 10 | NM | Y | N | NM | Hip surgery | Y | NM | Comparison of alone PENG (5 patients) and Combination with LFCN (5 patients) | LFCN block | N | N | POA | A combination of the PENG and LFCN block provides better reduction in pain scores than PENG block alone | N | NM | NM | NM | NM | NM | S | NM | NM | NM | NM | NM | S | NM |
| 50 | Sahoo RK | Sahoo RK, Jadon A, Sharma SK, Peng PW | Indian J Anaesth | Peri-capsular nerve group block provides excellent analgesia in hip fractures and positioning for spinal anaesthesia: A prospective cohort study | PCS | 20 | El | Y | N | N | Hip surgery | Y | Before SA | N | N | N | SA | Pos_A | Reduction in pain score (VAS) - a median drop of 6 to 7 points 30 minutes following the block both at rest and passive movements. Comfortable positioning during SA | N | B | 0.25 | 20 | D | Y | S | CL | LW | 2 to 5 | IN | LTM | S | Supine |
| 51 | Sandri M | Sandri M, Blasi A, De Blasi RA. | J Anesth | PENG block and LIA as a possible anesthesia technique for total hip arthroplasty | CS | 10 | Ad/ El | Y | N | N | Hip surgery | Y | Y | Combination of PENG with LIA | LIA | N | midazolam and fentanyl to improve acceptance of anesthesia and paracetamol, ketoprofen, and dexamethasone for postopertaive analgesia | IOA POA | Uneventful perioperative period and Reduction in pain score (NRS) postoperatively | N | LB | 0.25 | 40 | D | N | S | NM | NM | NM | IN | LTM | S | Supine |
| 52 | Santos O | Santos O, Pereira R, Cabral T, Lages N, Machado H. | J Anesth Clin Res | Is continuous peng block the new 3-in-1 | CR | 1 | Ad | Y | N | Y | Hip surgery | Y | Before GA | N | N | GA | Postoperative analgesia was 100 mg tramadol intravenously and 1 g of paracetamol bolus | IOA POA | Reduction in pain score (NRS) - rest and dynamic - to 2 after 8 h and 0 at 24 and 48 h | N | R | 0.5(S) + 0.5(S) + 0.2© | 15(S) + 20(S) +5ml/h © | D | N | S + C | CL | LW | 2 to 5 | IN | LTM | S + C | Supine |
| 53 | Sardesai AM | Sardesai AM, Biyani G | Indian Anaesthetists Forum | Pericapsular nerve group block: Innovation or just a fad? | EDITORIALS |  |  |  |  |  |  |  |  |  |  |  |  |  |  |  |  |  |  |  |  |  |  |  |  |  |  |  |  |
| 54 | Sidhu GK | Sidhu GK, Khatri K, Jindal S | Reg Anesth Pain Med | APENG vis-à-vis PENG | EDITORIALS |  |  |  |  |  |  |  |  |  |  |  |  |  |  |  |  |  |  |  |  |  |  |  |  |  |  |  |  |
| 55 | Singh S | Singh S | J Clin Anesth | Total hip arthroplasty under continuous pericapsular nerve group block (cPENG) in a high risk patient | CR | 1 | Ad | Y | N | Y | Hip surgery | Y | Solo | N | N | Propofol infusion and midazolam sedation | fentanyl (intraoperatively), acetaminophen (postoperatively) | IOA POA | uneventful intraoperative period. Reduction in VAS score (always less than 3) for 3 days. | N | B | 0.5,0.5,0.125 | 15(S) + 5ml/h© | N | N | S + C | CL | LW | NM | IN | NM | S + C | Supine |
| 56 | Singh S | Singh S, Singh S, Ahmed W. | A A Pract | Continuous Pericapsular Nerve Group Block for Hip Surgery: A Case Series | CS | 10 | Ad/ El | Y | N | N | Hip surgery | Y | Before SA | N | N | SA | 1 mg midazolam and 50 μg fentanyl | Pos_A IOA POA | Uneventful perioperative period and Reduction in pain score (VAS) to 1 to 3, 30 minutes after PENG block. Comfortable repositioning for SA (VAS was 1 or 2 during SA). | N | B | 0.25 | 20 (S) + 5 ml/h © | N | N | S + C | CL | LW | NM | IN | LTM | S + C | Supine |
| 57 | Singh S | Singh S. | J Clin Anesth | Advocating the use of continuous pericapsular nerve group (PENG) block to maximize its advantages | EDITORIALS |  |  |  |  |  |  |  |  |  |  |  |  |  | Limiting the volume of drugs can decrease the duration of a block which can be overcome by using a continuous infusion through a placed catheter. Use of catheter prolong the duration of analgesia. | Motor block in few cases | B | 0.25 | 10 (S)+ 5 ml/h© | N | N | S + C | NM | NM | NM | NM | NM | S + C | Supine |
| 58 | Talawar P | Talawar P, Tandon S, Tripathy DK, Kaushal A | Indian J Anaesth | Combined pericapsular nerve group and lateral femoral cutaneous nerve blocks for surgical anaesthesia in hip arthroscopy. | CR | 1 | Ad | Y | N | N | Hip surgery | Y | Before sedation | Combination - PENG and LFCN block | LFCN | midazolam and fentanyl sedation | N | IOA | Successful surgical anaesthesia | N | B , LD | 0.5,2 | 20 (10 +10) (PENG) + 12 (LFCN) | N | Y | S | CL | LW | 3 to 5 | IN | NM | S | Supine |
| 59 | Thallaj A | Thallaj A. | Saudi J Anaesth | Combined PENG and LFCN blocks for postoperative analgesia in hip surgery-A case report | CR | 1 | El | Y | N | Y | Hip surgery | Y | After GA | Combination - PENG and LFCN block | LFCN block | GA | fentanyl 2.5  mcg/kg (perioperatively ) and acetaminophen 8 mg/kg (postoperatively) | POA | Reduction in pain score (NRS) - rest and dynamic - to 0 (rest) for 24 h and 2 (rest) and 3 (dynamic) respectively at 36 and 48 h. | N | B | 0.25 | 30 | N | N | S | CL | LW | 2 to 5 | IN | LTM | S | NM |
| 60 | Tran J | Tran J, Agur A, Peng P. | Reg Anesth Pain Med | Is pericapsular nerve group (PENG) block a true pericapsular block? | EDITORIALS |  |  |  |  |  |  |  |  |  |  |  |  |  |  |  |  |  |  |  |  |  |  |  |  |  |  |  |  |
| 61 | Wyatt K | Wyatt K, Zidane M, Liu CJ. | Case Rep Orthop | Utilization of a Continuous Pericapsular Nerve Group (PENG) Block with an Opioid-Sparing Repair of a Femoral Neck Fracture in a Pediatric Patient | CR | 1 | P | Y | N | N | Hip surgery | Y | After GA | N | N | GA | N | IOA POA | Uneventful perioperative period and Reduction in pain score (NRS) to 2 and 3. FLACC and subjective pain scores were 0/10 for the ensuing 12 h. | N | B,R | 0.25,0.1 | 14 (S) + 6 ml/h© | N | N | S + C | CL | LW | 2 to 5 | IN | LTM | S + C | Supine |
| 62 | Wyatt KE | Wyatt KE, Pranav H, Henry T, Liu CJ. | J Clin Anesth | Pericapsular nerve group blockade for sickle cell disease vaso-occlusive crisis | CR | 1 | P | Y | N | Y | Pain management | Y | After Sedation | Combination — PENG and FN block | FN block | N | N | Analgesia | Reduction in pain score (NRS) to 0 after block. No additional analgesia needed. Patient was able to ambulate post procedure. | N | B | 0.25 | 16 | Dex | N | S | NM | NM | NM | NM | NM | S | Supine |
| 63 | Yamak Altinpulluk E | Yamak Altinpulluk E, Galluccio F, Salazar C, Espinoza K, Olea MS, Hochberg U, de Santiago J, Fajardo Perez M. | J Clin Anesth | Peng block in prosthetic hip replacement: A cadaveric radiological evaluation | CADAVERIC |  |  |  |  |  |  |  |  |  |  |  |  |  |  |  |  |  |  |  |  |  |  |  |  |  |  |  |  |
| 64 | Yu HC | Yu HC, Moser JJ, Chu AY, Montgomery SH, Brown N, Endersby RVW. | Reg Anesth Pain Med | Inadvertent quadriceps weakness following the pericapsular nerve group (PENG) block | CR | 2 | Ad/ El | Y | N | Y | Hip surgery | Y | One before and one after surgery | N | N | SA | oral and IV opioids | Pos_A IOA POA | Reduction in pain score — rest and dynamic | Inadvertent motor block | B | 0.5, 0.25 | 20 | D | Y | S | NM | NM | NM | IN | LTM | S | Supine |

| **Abbreviation used in the table** | **Stands for** |
| --- | --- |
| % | Percentage |
| Ad | Adult |
| B | Bupivacaine |
| C | Continuous infusion |
| CL | Curvilinear |
| CR | Case report |
| CS | Case series |
| D | Dexamethasone |
| Dex | Dexmedetomidine |
| El | Elderly |
| ESPB | Erector spinae block |
| FIB | Fascia illiaca block |
| FN | Femoral Nerve |
| GA | General anaesthesia |
| HH | High |
| h | Hour/Hours |
| IN | In the plane |
| IOA | Intraoperative analgesia |
| kg | kilogram |
| LA | Local anaesthetic |
| LB | Levo-bupivacaine |
| LD | Lidocaine |
| LESP | Lumbar erector spinae plane |
| LFCN | Lateral femoral cutaneous nerve |
| LIA | Local anesthetic infiltration |
| Lin | Linear |
| LTM | Lateral to medial |
| LW | Low |
| mg | mili gram |
| MHz | Mega hertz |
| ml | Mililiters |
| MTL | Medial to lateral |
| N | No |
| NM | Not mentioned |
| NRS | Numerical rating scale |
| NS | Normal saline |
| OME | Oral morphine equivalents |
| OUT | Out of plane |
| P | Paediatric |
| PCA | Pateint controlled analgesia |
| PCS | Prospective cohort study |
| PENG | Pericapsular nerve group |
| POA | Post-operative analgesia |
| Pos_A | Analgesia for Positioning |
| R | Ropivacaine |
| RCT | Randomized controlled trial |
| S | Single shot |
| SA | Spinal anaesthesia |
| VAS | Visual analogue score |
| Y | Yes |
